# Rule-Based vs. Behavior-Based Self-Deployment for Mobile Wireless Sensor Networks

**DOI:** 10.3390/s16071047

**Published:** 2016-07-07

**Authors:** Cristina Urdiales, Francisco Aguilera, Eva González-Parada, Jose Cano-García, Francisco Sandoval

**Affiliations:** Departamento de Tecnologia Electronica, ETSI Telecomunicacion, Campus de Teatinos s/n, University of Malaga, Malaga 29010, Spain; fjaguilera87@gmail.com (F.A.); gonzalez@uma.es (E.G.-P.); jcgarcia@uma.es (J.C.-G.); sandoval@uma.es (F.S.)

**Keywords:** mesh mobile sensor networks, Wireless sensor network (WSN), deployment, social potential fields (SPF), BDA

## Abstract

In mobile wireless sensor networks (MWSN), nodes are allowed to move autonomously for deployment. This process is meant: (i) to achieve good coverage; and (ii) to distribute the communication load as homogeneously as possible. Rather than optimizing deployment, reactive algorithms are based on a set of rules or behaviors, so nodes can determine when to move. This paper presents an experimental evaluation of both reactive deployment approaches: rule-based and behavior-based ones. Specifically, we compare a backbone dispersion algorithm with a social potential fields algorithm. Most tests are done under simulation for a large number of nodes in environments with and without obstacles. Results are validated using a small robot network in the real world. Our results show that behavior-based deployment tends to provide better coverage and communication balance, especially for a large number of nodes in areas with obstacles.

## 1. Introduction

Wireless sensor networks (WSNs) are formed by a set of spatially-distributed sensors that connect to each other. Each sensor node is formed by: (i) a micro controller; (ii) one or more sensors; and (iii) a wireless transceiver. These nodes may have some degree of autonomy. Their function is to monitor their physical environment and report to sink nodes, which redirect data to the appropriate destination. A key issue in WSNs is how to deploy the network as efficiently as possible in terms of coverage and energy efficiency. Deployment has a strong impact on connectivity and throughput [1]. Deployment of large WSNs presents a number of problems [2]. In many applications, networks have to be deployed over a large, potentially dynamic area, so predefining an optimal configuration may be difficult. Furthermore, if a node breaks or runs out of battery, part of the network might get isolated. WSNs usually rely on a mesh topology, which is more redundant, but also more robust and easier to expand and modify. Unfortunately, it is also hard to set up and maintain.

Deployment can be simplified if WSNs can reconfigure themselves. If a node fails, part of the network may adjust to fill the gap as long as nodes can reposition themselves and/or change its roles. Mobile WSNs (MWSNs) can modify their positions to reorganize the network on a needs basis [3]. MWSNs are useful both for self-deployment, as nodes can move towards their most adequate position, and self-repair, so live nodes can cover dead ones. In order to move autonomously, MWSNs may be deployed by multiple robots systems (MRSs). In this case, nodes also include motors and sensors, and their micro controllers run a basic control system in addition to the WSN code.

The main goal of MWSN deployment is to use as few nodes as possible to cover a given area and to extend the WSN lifetime as much as possible before batteries need to be replaced. A node battery life mostly depends on the amount of traffic that it is generating/routing, so some parts of the WSN will fail earlier than others. In these cases, robots reorganize their positions to keep it working [4]. Robot deployment requires autonomous navigation skills, but also coordination and localization techniques. GPS is not appropriate in certain environments due to occlusions and the multi-path effect [5]. Active localization is typically computationally less expensive than passive localization or tracking, so most MWSN systems rely on received signal strength (RSS)-based triangulation. Sometimes, a static beacon infrastructure is fixed for mobile nodes to locally position themselves [6,7]. In these cases, deployed nodes can act themselves as beacons for the rest [5,8].

There are two different strategies for autonomous self-deployment [9,10]. Deliberative systems build a model of the environment, including the relative locations of the nodes and events, to predict their best deployment positions. This methodology is frequently used for static deployment, where nodes are not expected to move afterwards and distribution needs to be optimized as much as possible [11]. In MWSNs, a master node gathers information about the whole network, makes all decisions and commands the other nodes where to move. This process is computationally expensive and usually involves techniques like genetic algorithms [12] or, in order to distribute the calculation, particle swarm optimization [13,14,15] and artificial bee colonies [16]. In order to reduce the computational complexity, deployment can be incremental, i.e., nodes are deployed one at a time, with each node making use of information gathered by the previously-deployed nodes to determine its ideal deployment location [5]. Even in this case, deployment requires specific hardware to support intense computation and clearly involves a higher traffic load, especially in the vicinity of the node running the optimization algorithm. Distributed implementations of deliberative algorithms may reduce computational load and provide a better traffic distribution up to some point [17].

Alternatively, reactive systems consider that local dispersion leads naturally to global dispersion, so each node makes decisions according to local factors, e.g., RSS. This approach involves a lower computational load and less intense, better distributed communication. In exchange, deployment is not necessarily optimized. Reactive systems are distributed by nature.

In this work, we evaluate two different reactive deployment approaches: rule-based deployment and behavior-based deployment. In rule-based deployment algorithms, nodes move following mutually exclusive rules until a termination condition is met: afterward, nodes stay in place [18,19]. In behavior-based deployment, each node presents a number of basic nuclear behaviors that are combined into an emergent motion action: a node stops when these behaviors reach an equilibrium, i.e., emergent motion is null. Examples of such algorithms are the well-known virtual potential and forces approaches [20,21,22]. Both rule-based deployment and behavior-based deployment present complementary advantages and drawbacks. Rule-based deployment may be adjusted to conform specific topologies, e.g., bus or ring networks, and to fit environment constraints, e.g., deploy along a land boundary. Furthermore, some nodes usually stop early during deployment, before their odometry-based localization is too affected by errors. Stationary nodes can be used as beacons for RSS-based localization for the rest [8]. However, these methods tend to return poorer coverage, and deployed networks are hard to recover when nodes start to fail. Furthermore, depending on the resulting topology, the load in the nodes may present significant variations. Behavior-based deployment is usually more popular in MWSNs, and there are many implementations in the field. They typically provide better coverage, and they can rearrange in case a node fails. However, nodes tend to move larger distances on average. Furthermore, localization in these cases is harder because there are no natural beacons: any node may move at any time during deployment depending on the behavior of its surrounding nodes.

The goal of our work is to compare a representative rule-based deployment algorithm with a behavior-based one. We have specifically chosen the backbone dispersion algorithm (BDA) [23] and the social potential fields (SPF) [24] because both are particularly fit to run on a robot swarm. These algorithms are introduced in Section 2. We have adapted BDA to run on obstacle-avoiding robots during deployment and SPF to preserve connectivity in the network, since it was originally designed for robot swarms. Our evaluation metrics are described in Section 3. Performance depends on the number of nodes in the network. One of the contributions of this work is that we have extended the Player/Stage environment, typically used to test robot behaviors, for network deployment. Hence, we can use the same code in simulations and real tests. Experiments with a large number of nodes have been performed under simulation. We have also validated our conclusions with a very reduced set of nodes in real environments. Networks this small are not fit to extract conclusions about deployment, but these tests confirm the simulation results and provide insight about the difficulties of real-world deployment. Our experimental setup is presented in Section 4.1. Then, results are presented in Section 4. Finally, conclusions are presented in Section 5.

In this work, nodes are robots equipped with a communication module, one or more sensors and a processing unit, plus onboard short-range sensors to prevent collisions.

## 2. Chosen Deployment Algorithms

The BDA [23] is a representative rule-based deployment algorithm, where a node moves randomly until it fulfills a termination condition. A node reasons in terms of how many nodes to which it can connect. BDA does not require localization information [25]. Connectivity is determined by RSS. Collisions between robots are avoided using onboard range sensors. Once a robot stops, it will not move again.

BDA [23] relies on creating a connected node structure, i.e., a backbone. It uses the following deployment rules. If a node is connected to:

- at least two robots and one of them belongs to the backbone: the robot keeps moving randomly and avoiding obstacles to spread the network as much as possible.
- a single robot that belongs to the backbone: to prevent loss of connectivity, the robot stops until it gets connected to another robot that travels nearby.
- at least a robot, but none of these robots belong to the backbone: the robot joins the backbone, stops and notifies its change of status to the rest of the network.
- no robot: the robot moves backwards until it finds some robot with which to connect.

This algorithm is simple and requires little communication among nodes. This implementation is not fit for self-healing because, when nodes stop, they are not supposed to move anymore. Furthermore, it is not adequate for hierarchical networks, where nodes adopt roles depending on their location and connectivity.

We have chosen an implementation of the SPF algorithm to represent behavior-based deployment algorithms. This algorithm was originally proposed for swarm robots [24], but we have adapted it to mobile WSN deployment. SPF relies on modeling a set of simple attraction and repulsion forces corresponding to goals or constraints, e.g., avoiding collisions, traveling as far as possible from other nodes, etc. We have added an additional behavior for deployment: preserve connectivity with at least another node. All of these forces are combined into an emergent motion vector. Nodes stop moving when this motion vector is null. In practice, nodes stop when this vector is under a threshold $f_{u}$ to reduce sensitivity to noise, errors and small changes in the environment. Deployment finishes when all robots stop moving. However, any major change in its input instance (e.g., a nearby node fails or moves) may make a robot move again.

In our implementation of SPF, each robot *i* is affected by three types of forces.

- Repulsion force(s) $f_{r1}\left(r_{i,j}\right)$ that pushes the robot away from nearby robots or obstacles to prevent collisions.
- Repulsion force(s) $f_{r2}\left(r_{i,j}\right)$ that aims at expanding the network by keeping robots away from each other.
- An attraction force (clustering force) $f_{c}\left(r_{i,j}\right)$. Attraction grows along with $r_{i,j}$ to prevent the loss of communication.

$r_{i,j}$ is the distance between robots *i* and *j*. Unlike in BDA, in SPF, we need to estimate robot locations to calculate their relative distances. These locations are always available under simulation. In real rests, locations are estimated using the robots odometry, assuming that they move at a constant speed from their original position, until some of them stop moving. Then, these robots are used as beacons for RSS-based trilateration [8]. The trilateration method in [8] would need to be adapted for our specific radiofrequency (RF) chipset for real tests. However, since our number of real robots is so reduced, we simply assume that there will be localization errors. For larger swarms in real tests, it could be advisable to use a transceiver with integrated location, as presented in [26].

As proposed in [24], all forces are estimated for every node *j* in the vicinity of *i* and then combined into an emergent motion vector. Force parameters are heuristically set, depending on the hardware features of the robots, mainly size and RF chipset. In this work, we have heuristically obtained the following parameters from tests with our real robots:

(1) $$f_{r1}\left(r_{i,j}\right)=-\frac{0.01}{{\left(r_{i,j}\right)}^{8}}$$

This value has been adjusted so that robots farther than one meter do not affect each other. However, since we have set $f_{u}$ to 0.5 to avoid oscillations, this force only operates when robots are closer than 60 cm from each other. The same equation applies to robots and static obstacles.

We have adjusted the clustering force parameters to affect robots when they are at least 1.5 m away:

(2) $$f_{c}\left(r_{i,j}\right)=-\frac{20}{{\left(r_{i,j}\right)}^{6}}+\frac{2}{{{(}_{i,j})}^{0.2}}$$

Finally, the second repulsion force is adjusted to keep a distance of approximately two meters between each of two robots:

(3) $$f_{r2}\left(r_{i,j}\right)=-\frac{60}{{\left(r_{i,j}\right)}^{7}}$$

We use these parameters obtained from real robots also in simulations. The overall behavior of the network is that nodes tend to move away from each other in every direction, avoiding collisions with each other and any obstacle in the way and preserving connectivity to cover an area as large as they can. No specific structure is constructed in this case.

## 3. Evaluation Parameters

Evaluation of network deployment requires the definition of a set of quality parameters.

Coverage is a typical quality measure in networks. Gage [27] defines three types of coverage: (i) blanket coverage, focused on achieving a static arrangement of nodes that maximizes the total detection area; (ii) barrier coverage, focused on minimizing the probability of undetected penetration through the barrier; and (iii) sweep coverage, equivalent to a moving barrier. The most fitting one for our work is blanket coverage, i.e., any point of the region is sensed by at least one sensor. If a single node *i* covers a round area $A_{i}$, given *N* sensors in a full area *A*, coverage *C* can be calculated as the ratio between the union ∪ of all $A_{i}$ and *A*:

(4) $$C=\frac{\cup_{i=1...N}A_{i}}{A}$$

Equation (4) can be modeled using a probabilistic grid of *M* cells [28], where we assign to each cell *i* a probability $p_{i}$ of detecting an event on that specific cell. Several nodes might detect an event at cell *i* independently, so we obtain $p_{i}$ in terms of the probability of an event going undetected at cell *i* (${\bar{p}}_{i}$):

(5) $$p_{i}=1-{\bar{p}}_{i}=1-\prod_{N}\left(1-p_{ij}\right)$$

*N* being the number of nodes and $p_{ij}$ being the probability of node *j* detecting an event at cell *i*.

Then, coverage *C* is obtained as:

(6) $$C=\sum_{i=1}^{M}\frac{p_{i}}{M}$$

Energetic efficiency is another accepted quality parameter in mesh networks. There are two different energy costs in these networks: (i) deployment and (ii) maintenance. Deployment costs mostly depend on two parameters [29]: distance *d* traveled by each node to its final location; and time *t* to reach its final location. After deployment is complete, energy cost is usually related to how homogeneously nodes are distributed. Uniformity *U* for *N* nodes can be defined as:

(7) $$U=\frac{1}{N}\sum_{i=1}U_{i}$$

(8) $$U_{i}={\left(\frac{1}{K_{i}}\sum_{j=1}^{K_{i}}{\left(r_{i,j}-{\bar{r}}_{i}\right)}^{2}\right)}^{1/2}$$

$K_{i,j}$ being the number of nodes in the vicinity of node *i*, $r_{i,j}$ being the distance between nodes *i* and *j* and ${\bar{r}}_{i}$ being the average distance between node *i* and its closest nodes. A high *U* usually leads to higher network lifetime because traffic load tends to be better distributed among nodes.

The network lifetime also depends on energy consumption.

Energy consumption depends on many factors, including separation among nodes, routing strategies and packet losses. In order to evaluate them indirectly, energetic efficiency can be roughly approximated by the average power that nodes require to send a message to the network $\bar{P}$:

(9) $$\bar{P}=\frac{1}{N}\sum_{i=1}^{N}{\bar{P}}_{i}$$

${\bar{P}}_{i}$ being the average power required at node *i* to send a message to the rest of the network, obtained as:

(10) $${\bar{P}}_{i}=\frac{1}{N}\sum_{i=1}^{N-1}P_{ij}$$

$P_{ij}$ being the power required to send a message from node *i* to node *j*.

In networks where messages need to be retransmitted through *k* nodes, it is necessary to add the power required for each hop:

(11) $$P_{ij}=P_{i1}+...+P_{ik}$$

In this work, we are also going to measure unbalance in the network after deployment as the ratio between the number of messages that the most loaded node is routing with respect to the number of messages transmitted by the least loaded node. When mesh networks are not well balanced, some nodes run out of battery much earlier than others and full areas of the network may get disconnected. Unbalance is also related to the deviation in the number of routed packages per sent message in the network ($\sigma Msg_{Tx}$). A large deviation means that some nodes are routing far more messages than others.

## 4. Experiments and Results

### 4.1. Experimental Setup

In order to test our system, we worked both with real robots and under simulation. In all of our tests, robots were originally placed together in the center of the test environment, and then, they were allowed to move until deployment was complete, i.e., the robots are not moving anymore. This work focuses on deployment, so we use a simple routing mechanism, closest neighbor: a robot only retransmits a message if it is closer to the destination robot than the one from which it received the message. This routing is used to measure traffic load on the nodes under working conditions. In all of our simulations, nodes transmit at −10 dBm, corresponding to our measures using the real robots. In closest neighbor routing, nodes may retransmit the same package more than once in case of losses. Consequently, results depend on the loss probability in the network.

**Figure 1:**
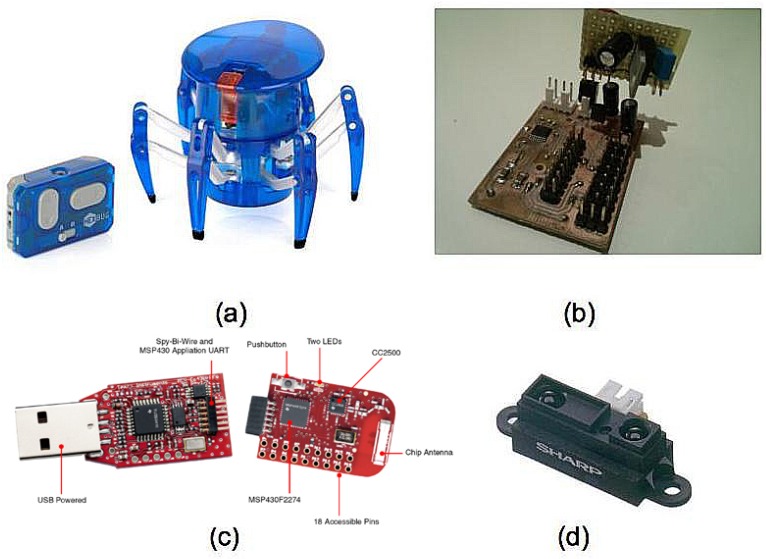
Node hardware structure: (**a**) Hexbug© spider; (**b**) our control board; (**c**) an EZ430-RF2500 kit; (**d**) a SHARP IR range sensor.

We built a small number of robots for real-world experiments (Figure 1). We reused off-the-shelf inexpensive robot toys for fast prototyping (Figure 1a). These toys have a number of drawbacks; mainly, they cannot carry much weight; they are unsuitable for rough terrain; and above all, legged robots do not provide odometry, so localization was purely based on speed calibration for time-based displacement estimation and RSS trilateration. Still, this approach allowed us to build several units for a reduced cost. Our control board mainly consisted of an H-bridge for 2-motor control (robot heading and forward/backward displacement), feeding, protection and conditioning circuitry (Figure 1b). We attached an EZ430-RF2500 unit to each robot (Figure 1c). These units include a MSP430F2274 microcontroller and a CC2500 RF chip, both from Texas Instruments Inc. (Dallas, Texas, USA). The system includes SimpliciTI, a propriety low-power network stack to enable off-the-box 2.4-GHz wireless networks. These robots may avoid each other using RSS to measure proximity, but there might be obstacles in the environment, as well, so we finally added an infrared range sensor (Figure 1d) from SHARP (Osaka, Japan) that works between 10 and 80 cm. All experiments with these robots were run over flat surfaces due to hardware limitations.

Even though the number of robots is too reduced to extract conclusions about deployment, using real robots allows us to: (i) set all hardware-dependent parameters in our equations; and (ii) validate that the emerging behavior of the real robots is similar to the behavior of the simulated ones.

**Figure 2:**
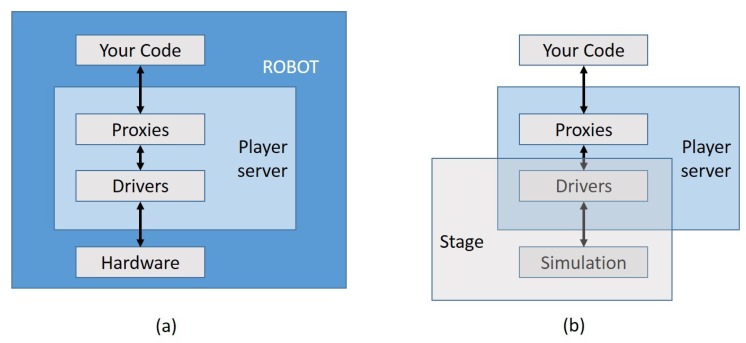
Player/Stage: connecting to: (**a**) a real robot; (**b**) a simulated robot.

We used the freeware Player environment [30] to support our control system. Player offers a client/server model for robot control that can be programmed in several languages. Player has built-in support for many different systems and sensors, and it is fairly simple to include ad hoc robots like ours (Figure 2a). Player may run on any computer with a network connection to the robot and supports multiple concurrent client connections, so it is adequate for an MRS. Furthermore, Player works with 2D and 3D freeware simulation ends (Stage and Gazebo, respectively). As commented, the performance of deployment algorithms is largely affected by scale: some may work perfectly with a small number of nodes and perform poorly when the network is large. Hence, most testing was done under simulation. In these cases, we used the Stage multiple robot simulator. Stage devices present a standard Player interface, so replacing a real robot with a simulated one is almost transparent to the control system [31] (Figure 2b). Using real robots also allowed us to check this Player/Stage feature.

### 4.2. Simulations

We have conducted two types of simulations, for medium and large networks in environments of 6 and 15 m^2^, using 20 and 100 nodes, respectively, with and without obstacles. Deployment is assumed to be finished when no robot is moving anymore. Table 1 shows the simulation results both for BDA and SPF. The results present some variation each time a test is run, but they basically depend on *N*, the obstacle layout (Obst) and the deployment algorithm (Dply). All tests for BDA and SPF are run in the same environments for comparison. Evaluation parameters include the average time to reach deployment position $\bar{t}$, the average distance traveled per node $\bar{d}$, coverage *C* (Equation (3)), uniformity *U* (Equation (7)), average power in the network $\bar{P}$ (Equation (9)), deviation in the number of routed messages per sent message per robot after deployment ($\sigma Msg_{Tx}$) and unbalance, calculated as the ratio between the number of messages routed per sent package by the most and the least loaded nodes after deployment (nodes routing zero messages are not considered in this calculation). A high unbalance means that some nodes will fail much earlier than others; hence, the network life will be shortened, and self-healing will often be required to extend it.

**Figure 3:**
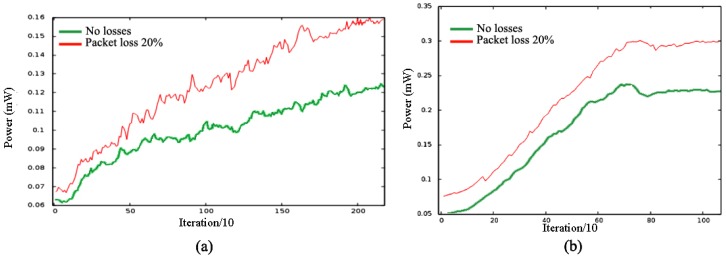
$\bar{P}$ during deployment: (**a**) BDA; (**b**) SPF.

$\bar{P}$ changes during deployment because nodes start to increase their relative distances when they move. Figure 3a shows a scenario for N=100 using BDA deployment. As robots start to deploy, power in the network grows until the final configuration is reached. Figure 3b shows another scenario for N=100 using SPF deployment. Both plots are similar, but in this case, the curve slope is steeper and the function stabilizes faster, typically around a higher ${\bar{P}}_{m}$ value than in BDA. This effect in explained in the following subsections. Table 1 presents the final ${\bar{P}}_{m}$ value in all cases.

If we increase the packet loss probability (random losses), $\bar{P}$ evolves similarly in both cases, but reaches a higher value depending on the loss probability. Figure 3 shows the same scenarios for a loss probability equal to 20%. The rest of the measured parameters, e.g., $\bar{t}$, $\bar{d}$, *C*, *U* and even $\sigma Msg_{Tx}$, reach similar values in all our scenarios using closest neighbor routing.

#### 4.2.1. BDA

**Figure 4:**
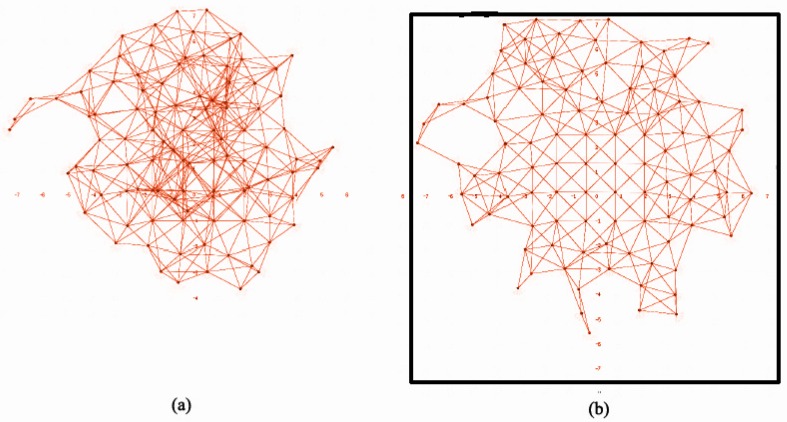
BDA for 100 robots: (**a**) (faulty) trapped robots; (**b**) (correct) shadow areas in boundaries.

In BDA simulations, deployment time $\bar{t}$ and the average distance covered by a node $\bar{d}$ grow with the size of the environment, as expected (Table 1). It can be observed that $\bar{d}$ grows faster than $\bar{t}$, especially when the number of nodes *N* involved is higher. Similarly, $\bar{t}$ and $\bar{d}$ grow when there are obstacles in the environment. The shape of obstacles affects the structure of the backbone, and all nodes have to adapt their positions accordingly. *U* in BDA is not particularly high for any number of nodes because BDA favors connectivity rather than uniformity. There are two frequent uniformity problems in BDA deployment, particularly in simulations with many nodes. In Figure 4a, some robots got trapped in the center of the network due to local minima. This provokes high density node areas and, hence, poorer coverage. In Figure 4b, the deployment is correct; however, there are uncovered areas, and the distribution is not homogeneous. This happens because in BDA, robots tend to conform to lines, so boundaries are poorly covered. Furthermore, line formations are particularly weak with respect to node failures, which might lead to disconnections of a mild number of nodes.

It can be observed that $\sigma Msg_{Tx}$ is quite similar in all cases because nodes are usually a hop away from the backbone. However, messages may need to hop more or less along the backbone depending on how far transmitter and receiver nodes are. Hence, the network is typically unbalanced, more so when *N* is high. We can see in Table 1 that when N=100, the most loaded node may transmit up to 30 times more packages than the least loaded one. Furthermore, coverage in these cases is not optimal either and decreases significantly with *N*. This is probably related to the need for preserving a backbone and keeping all of the other nodes near this backbone.

**Figure 5:**
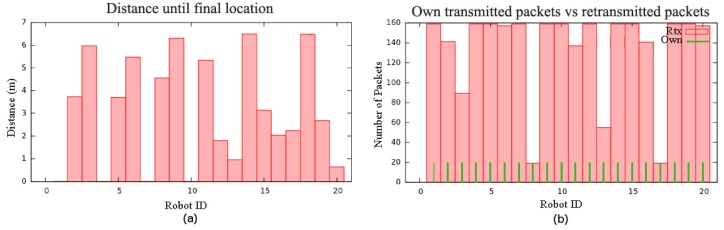
BDA deployment for 20 robots in an environment without obstacles: (**a**) *d* for every robot; (**b**) retransmission load.

A second effect of keeping a backbone is a large deviation in *d*: nodes joining the backbone first stop early during deployment, whereas others may need to travel significantly longer distances. Figure 5a shows *d* for each robot in N=20. Node 20, for example, travels less than one meter, whereas Node 14 travels almost seven meters.

Finally, we can observe that, after deployment, the ratio of retransmitted packets for each robot with respect to its own transmitted packets changes significantly from one robot to the next, and, in most cases, is quite large. As a general rule, robots on the network boundaries retransmit less packages than the rest, although robots in very crowded areas may also have a lower retransmission rate because nearby robots share their load. Figure 5b shows the number of messages routed per message transmitted in a 20-node scenario. It can be observed that the load is not well balanced. Robots on the boundaries of the coverage area typically route less messages than the rest. This also happens in areas with a higher robot density, where packets may be routed by one or another depending on their proximity to the destination robot. This unbalance tends to grow with the number of nodes (Table 1).

#### 4.2.2. SPF

**Figure 6:**
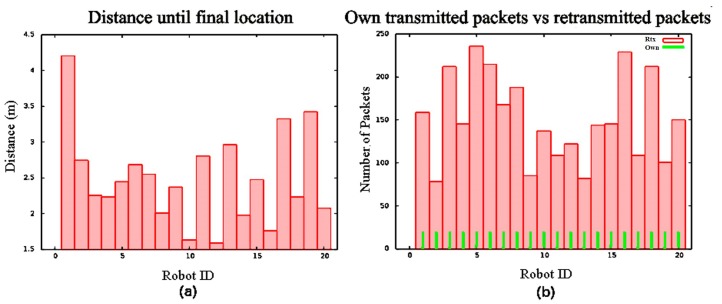
SPF deployment for 20 robots in an environment without obstacles: (**a**) *d* for every robot; (**b**) retransmission load.

Table 1 shows the results for SPF deployment in the same scenarios as in the previous BDA deployment. In this case, $\bar{t}$ and $\bar{d}$ also grow with the number of nodes, and there are slight differences in both parameters in environments with and without obstacles. However, in this case, robots deploy faster and travel less distance on average in every scenario. Figure 6a shows *d* for each robot in N=20. In this test, nodes travel between 1.7 and 4.3 m. It can be observed that distance grows progressively depending on the network expansion ratio, i.e., nodes tend to locate themselves in circles around their departure point. In SPF, $\bar{t}$ is less sensitive to *N*, and $\bar{d}$ is less sensitive to obstacles, especially for large *N* values. SPF does not preserve any type of structure, and nodes may move at any time to adapt to the input conditions. Hence, it is more flexible than BDA.

Flexibility in SPF also has an impact on coverage *C* and uniformity *U*. Robots are distributed in a more uniform way, and, hence, coverage is better. Furthermore, *C* is less sensitive to the obstacle layout in the SPF deployment. In BDA simulations, especially for a large number of nodes, *C* decreased below 60%. In the SPF deployment, it is above 90% in all cases.

Table 1 shows that $\bar{P}$ and $\sigma Msg_{Tx}$ are larger in SPF than in BDA simulations. Although the difference is not significant for smaller networks, it is noticeable for 100 nodes. This potential drawback is actually due to a better node distribution. $\bar{P}$ is larger because the covered area is larger as well, and also because all robots tend to keep a minimum distance from each other. In the BDA deployment, $\bar{P}$ is generally lower, but its deviation is larger. The lack of a basic infrastructure also leads to larger $\sigma Msg_{Tx}$ values in SPF, but, in exchange, it offers better balance: in our simulations, the most loaded node in the work case scenario routes approximately 10 times more packages than the least loaded one. This can also be observed in Figure 6b. After SPF deployment, nodes route more packages than in Figure 5b. However, the load is better distributed.

**Figure 7:**
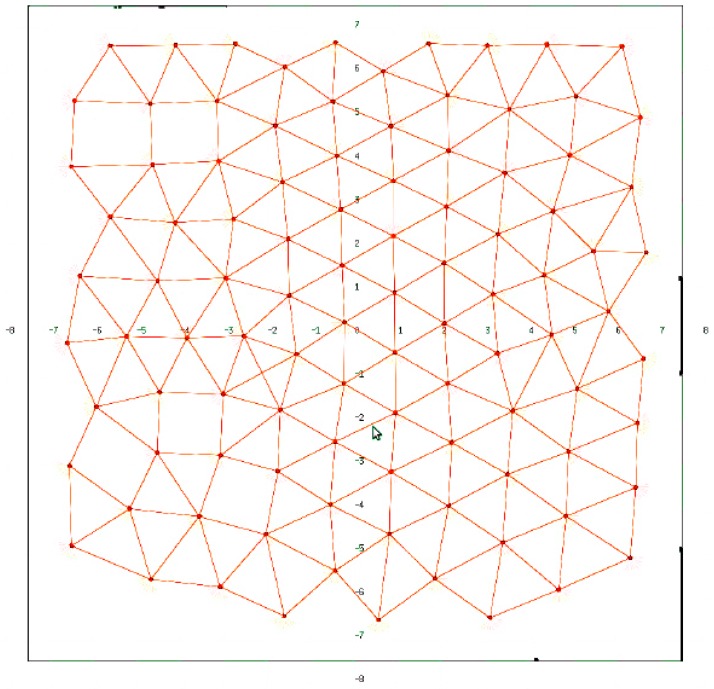
SPF deployment for 100 robots in an environment without obstacles.

Figure 7 shows the final deployment of a 100 node network in an environment without obstacles. All simulations in these environments return similar results. We can observe that nodes spread quite uniformly over the whole area.

**Figure 8:**
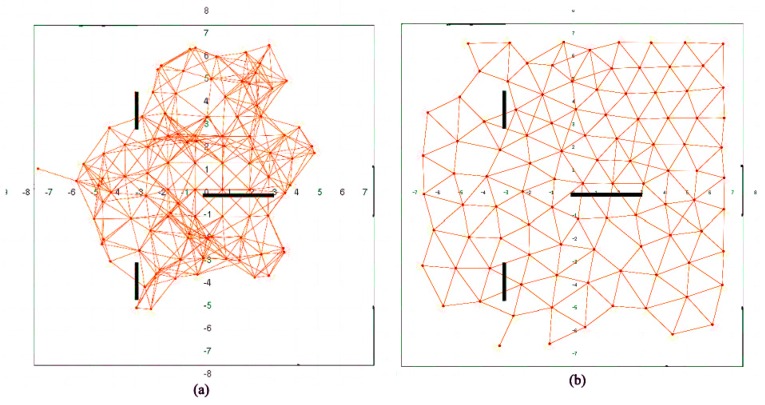
Deployment for 100 robots in an environment with obstacles using: (**a**) BDA; (**b**) SPF.

Deployment differences between both algorithms are more visible in environments with obstacles. Figure 8 shows a sample environment with obstacles. After BDA deployment, nodes tend to wrap themselves around obstacles to preserve their backbone structure. This leads to poorer coverage, lower uniformity and larger unbalance. Deployment results with SPF are similar to those in areas without obstacles (Figure 6): nodes simply try to avoid obstacles and reach equilibrium according to their defined behaviors.

**Figure 9:**
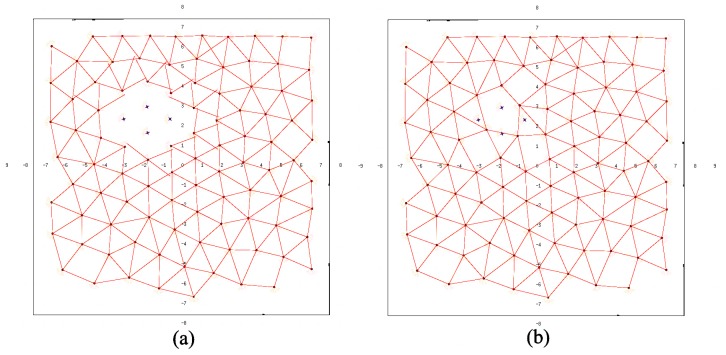
Self-healing using SPF for 100 robots in an environment with obstacles: (**a**) failure of four nodes; (**b**) reconfigured network.

Finally, Figure 9 shows a self-healing test. This experiment can only be performed with SPF because nodes in BDA are expected to keep their positions after deployment. In SPF, when one or several nodes fail, the equilibrium conditions for their neighbors are not met anymore, so surrounding nodes readjust their positions on a needs basis until equilibrium is reached again. Rearrangement may increase $\bar{P}$ and $\sigma Msg_{Tx}$ depending on the number of failed nodes, but *C*, *U* and unbalance tend to be preserved.

### 4.3. Tests with Real Robots

We also tested BDA and SPF deployment in the real world. As mentioned above, we used the same software in Player, but replaced our simulated robots in the Stage environment with the ones using the robots in Figure 1. Since we had a very reduced number of robots, these tests are not adequate to evaluate deployment itself, but rather to check how real-world conditions would affect the results on which we have already commented. The most important errors in the real world for our problem are: (i) lost messages; (ii) variations in RSS; (iii) localization errors. We have constrained our tests to structured indoor environments due to hardware limitations, so no additional considerations are required. The coverage area is surrounded by planar obstacles (“walls”) that robots can detect with their onboard sensor.

**Figure 10:**
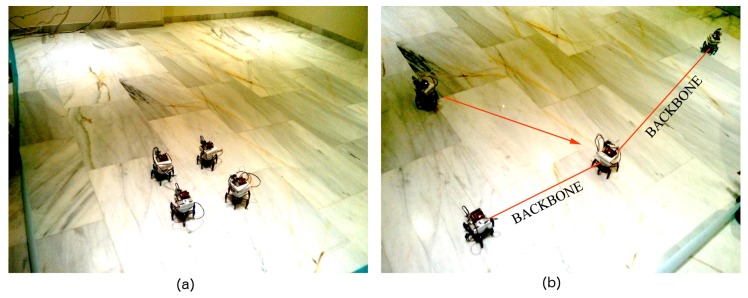
Deployment for four robots in a real environment using BDA: (**a**) initial configuration; (**b**) final configuration.

Figure 10 shows the BDA deployment of a four-node network. BDA is not sensitive to localization errors because it does not use position information. However, since it relies on RSS information, it is sensitive both to message loss and variations in RSS. In the first case, sometimes, robots decide to join the backbone too soon, and the deployment is incomplete (Figure 10b). Variations in RSS because of multiple reasons may have the same effect or also prevent nodes from joining the backbone when they should. In this case, deployment time grows. Deployment can also be delayed if nodes are originally positioned close to the walls, as reported in simulations with obstacles.

**Figure 11:**
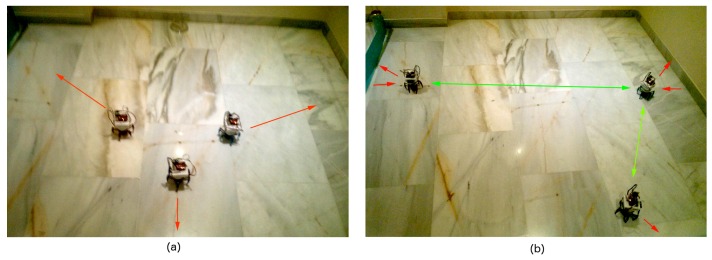
Deployment for three robots in a real environment using SPF: (**a**) initial configuration; (**b**) final configuration.

Figure 11 shows the SPF deployment of a three-node network. SPF is sensitive to localization errors because each robot takes into account the positions of those nearby when it moves. As mentioned above, in our legged robots, localization is a combination of speed-based position estimation and RSS triangulation. Both sources of information are quite noisy, so deployment does not work as well as under simulation. Still, since our tests included only a small number of nodes and, consequently, the deployment areas were reduced, the accumulated localization errors were not too large.

Table 2 shows our evaluation parameters in these tests. They validate our conclusions from simulation tests: SPF deployment is faster with respect to BDA, and the resulting coverage and uniformity are better (Figure 11b). As a result, unbalance and $\sigma Msg_{Tx}$ are lower. In exchange, $\bar{P}$ is larger in the SPF scenario, and robots travel a greater distance. Furthermore, in larger scenarios, localization would need to be improved. In both tests, coverage is limited. To increase this parameter, more robots would be needed.

**Figure 12:**
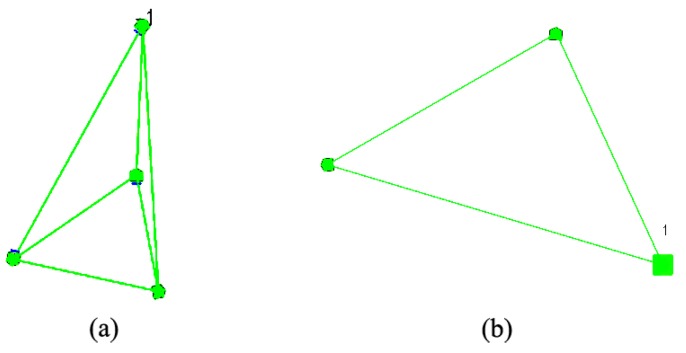
Simulation results for: (**a**) four robots using BDA; (**b**) three robots using SPF.

To validate our methodology even for such a limited number of robots, we have run the same scenarios from these real tests under simulation. Figure 12 shows the deployment results. It can be observed that both layouts are very similar to the ones in Figure 10b and Figure 11b.

## 5. Conclusions

This work has presented an experimental evaluation of rule-based (BDA) and behavior-based (SPF) reactive deployment algorithms for mobile WSNs. Both algorithms have been extensively tested under simulation in environments with and without obstacles for different numbers of nodes. Conclusions extracted from simulations have been validated in a real environment using a very reduced number of nodes. These tests also provided insight about the effect of noise and errors in the evaluated parameters. However, results in real environments cannot be conclusive unless a much larger number of nodes is involved in the tests.

The main conclusions of our evaluations are the following ones. SPF provides faster and more uniform deployment and a better coverage. Furthermore, the network load is better balanced after deployment in this case, meaning that although some nodes transmit more messages than others, unbalance is not as large as after BDA deployment. A better balance leads to a longer network life. Average power and $\sigma Msg_{Tx}$ after deployment are actually greater in SPF, mostly because nodes are more separated. However, these parameters may change from one node to the next, contributing to unbalance. SPF deployment also adapts better than BDA to areas with obstacles. All of these effects become more significant in large networks. BDA, however, is less dependent on localization and may offer better results if deployment needs to follow a set of fixed rules.

In brief, SPF seems more suitable for deployment in large, dynamic, unstructured areas, as long as node localization is reliable. Future work will focus on extending this study to hierarchical WSNs.

## Figures and Tables

**Table 1:** Deployment of different mesh networks using BDA and SPF.

*N*	Dply	Obst	$\bar{t}$	$\bar{d}$	*C*	*U*	$\bar{P}$	$\sigma Msg_{Tx}$	Unbalance
20	BDA	N	2 min 3 s	2.140	79.33%	0.578	0.052	34.8 %	6.73
20	BDA	Y	3 min 8 s	3.218	76.78%	0.574	0.051	32.5%	4.46
100	BDA	N	6 min 48 s	30.077	58.13 %	0.533	0.125	34.7 %	28.76
100	BDA	Y	7 min 31 s	40.145	59.86 %	0.545	0.11	34.7%	33.36
20	SPF	N	1 min 6 s	1.698	95.11 %	0.706	0.071	32.7%	2.62
20	SPF	Y	1 min 22 s	2.078	99.55 %	0.783	0.080	36.9%	4.6
100	SPF	N	2 min 38 s	18.499	93.67 %	0.727	0.225	42.8%	10.3
100	SPF	Y	2 min 57 s	19.57	90.84 %	0.711	0.215	42.7%	9.75

Dply: Deployment algorithm. Backbone deployment (BDA) and social potential field (SPF); Obst: Presence of obstacles in the scenario.

**Table 2:** Deployment of real mesh networks using BDA and SPF.

*N*	Dply	Obst	$\bar{t}$	$\bar{d}$	*C*	*U*	$\bar{P}$	$\sigma Msg_{Tx}$	Unbalance
4	BDA	N	40 s	0.91	52%	0.57	0.025	37%	3
3	SPF	N	30 s	1,12	59.7 %	0.79	0.041	27.2%	2

Dply: Deployment algorithm. Backbone deployment (BDA) and social potential field (SPF); Obst: Presence of obstacles in the scenario.

## Abbreviations

The following abbreviations are used in this manuscript:

BDA	backbone dispersion algorithm
SPF	social potential fields
RSS	received signal strength
RF	Radiofrequency
